# Characterization of the Healthy Ocular Mycobiome of Purebred Lusitano Horses Using Oxford Nanopore Long-Read Sequencing

**DOI:** 10.3390/ani16182913

**Published:** 2026-09-16

**Authors:** Mariana Torres Magalhães, Maria Eduarda Abreu, Mónica Nunes, Pedro Pascoal, Marcelo Pereira, Ricardo Dias, Teresa Rosa, Esmeralda Delgado, Manuela Oliveira, Luís Pardon Lamas

**Affiliations:** 1 Center for Interdisciplinary Research in Animal Health (CIISA), Faculty of Veterinary Medicine, University of Lisbon, Pólo Universitário da Ajuda, 1300-477 Lisbon, Portugal; eduardabreu.eq@gmail.com (M.E.A.); teresarosa@fmv.ulisboa.pt (T.R.); esmeralda@fmv.ulisboa.pt (E.D.); moliveira@fmv.ulisboa.pt (M.O.); llamas@fmv.ulisboa.pt (L.P.L.); 2 Hospital Escolar de Equinos, Faculty of Veterinary Medicine, University of Lisbon, Pólo Universitário da Ajuda, 1300-477 Lisbon, Portugal; 3 cE3c—Centre for Ecology, Evolution and Environmental Changes & CHANGE—Global Change and Sustainability Institute, Faculty of Sciences, University of Lisbon, Campo Grande, 1749-016 Lisbon, Portugal; msnunes@ciencias.ulisboa.pt (M.N.); pfpascoal@fc.ul.pt (P.P.); rpdias@fc.ul.pt (R.D.); 4 BioISI—Biosystems and Integrative Sciences Institute, Faculty of Sciences, University of Lisbon, 1749-016 Lisbon, Portugal; 5 Associate Laboratory for Animal and Veterinary Sciences (AL4AnimalS), 1300-477 Lisbon, Portugal

**Keywords:** equine ophthalmology, ocular mycobiome, Oxford Nanopore sequencing, Purebred Lusitano horse

## Abstract

The ocular surface represents a unique microbial ecosystem continuously exposed to environmental microorganisms; however, the fungal component of the healthy equine ocular microbiome remains poorly characterized. This study aimed to characterize the ocular surface mycobiome of 12 clinically healthy Purebred Lusitano horses from three geographical regions in Portugal kept in two housing systems using Oxford Nanopore long-read sequencing and conventional fungal culture. Sequencing revealed a highly diverse fungal community with marked inter-individual variability. The most abundant genera were *Wallemia*, *Podosphaera*, *Debaromyces*, *Aspergillus*, *Filobasidium*, *Kurtzmaniella*, *Metschnikowia*, *Malassezia* and *Spathaspora*. Patterns of fungal community composition suggested that local environmental conditions may contribute to the observed inter-individual variability. Genera commonly associated with equine fungal keratitis, including *Aspergillus*, *Fusarium* and *Penicillium*, were detected in healthy horses irrespective of housing system. Overall, the healthy equine ocular surface was characterized by a diverse mycobiome dominated by environmentally associated fungi. There findings provide a baseline for future studies investigating ocular microbial dysbiosis, environmental determinants of fungal community structure, and equine ocular health.

## Simple Summary

The microbiome of the ocular surface is composed of a large number of microorganisms dominated by bacteria, fungi and viruses. The fungal community present on the ocular surface is an important component of eye health but remains poorly characterized in horses. The objective of this study was to characterize the mycobiome of the ocular surface of clinically healthy Purebred Lusitano horses housed under different environmental conditions using a DNA-based sequencing approach, complemented with traditional fungal cultures. The most abundant fungal genera identified by sequencing included *Wallemia*, *Podosphaera*, *Debaryomyces*, *Aspergillus*, *Filobasidium*, *Kurtzmaniella*, *Metschnikowia*, *Malassezia* and *Spathaspora*. Conventional culture most frequently isolated *Candida*, *Mucor* and *Aspergillus*. Fungal genera commonly associated with equine fungal keratitis, including *Aspergillus*, *Fusarium* and *Penicillium*, were detected in horses from both housing environments. DNA-based sequencing provided relevant data on the ocular fungal mycobiome of healthy horses, which can be used as a valuable complementary tool for studying fungi associated with ocular health and disease.

## 1. Introduction

The ocular surface harbors a complex microbial community composed by bacteria, fungi, viruses and other microorganisms that coexist with the host in a dynamic ecological balance [1]. In healthy eyes, the ocular microbiota contributes to surface homeostasis through interactions with the host immune system and competition with potential pathogens. Although bacteria constitute the predominant microbial component of the equine ocular surface, fungi are also consistently detected and form the ocular mycobiota, a fungal community that is thought to be strongly influenced by continuous environmental exposure [2,3]. The ocular mycobiota exists in equilibrium with the host immune system [4], interacts with the resident bacterial microbiota [5], and may contribute to colonization resistance by competing with opportunistic pathogens for nutrients and ecological niches [6].

Despite its potential biological and clinical importance, the healthy equine ocular mycobiota remains poorly characterized. Most available studies have relied on conventional fungal culture, a technique that detects only viable fungi capable of growing under laboratory conditions and therefore provides an incomplete representation of fungal diversity [7]. Using culture-based methods, previous studies have consistently identified *Aspergillus* sp., *Penicillium* sp., *Cladosporium* sp., *Alternaria* sp., *Fusarium* sp., *Trichoderma* sp., *Scopulariopsis* sp., *Candida* sp., *Cryptococcus* sp. and *Rhodotorula* sp. among the fungi isolated from healthy equine eyes [4,5,8,9,10]. Some of these fungi are regarded as environmental saprophytes, whereas others are opportunistic pathogens capable of causing ocular disease following disruption of the corneal epithelial barrier [11].

The composition of the ocular mycobiota is influenced by multiple environmental and management factors, including geographical location, climate, season, bedding material, forage, and housing conditions [4,8,12,13]. Horses housed in stables may experience greater exposure to airborne dust, hay and bedding-associated fungi than pasture-kept horses, potentially favoring fungal colonization [9,14,15]. In contrast, Walsh et al. [16], using next-generation sequencing (NGS), reported greater fungal diversity in pasture-kept horses than in stabled animals, highlighting the influence of environmental exposure on fungal community composition. Moreover, other studies have found no significant effects of season, climatic conditions, age, sex or breed on the frequency of fungal isolation in healthy horses [4,6,15], suggesting that the factors shaping the equine ocular mycobiota remain incompletely understood.

Conventional fungal culture remains the reference method for diagnosing fungal infections because it allows recovery of viable organisms for phenotypic identification and antifungal susceptibility testing [17,18]. Advances in molecular diagnostic techniques have increasingly complemented conventional microbiological methods in both human and veterinary ophthalmology [19]. Unlike culture-based approaches, which rely on microbial growth under laboratory conditions, next-generation sequencing, particularly long-read sequencing using Oxford Nanopore Technology, identifies fungi based on their DNA rather than phenotypic characteristics, providing substantially greater taxonomic resolution and revealing fungal taxa that remain undetected by conventional culture [7,16,19,20,21].

Consequently, NGS has become a powerful tool for investigating complex microbial communities and expanding our understanding of the healthy ocular mycobiome.

Characterization of the healthy ocular mycobiota is particularly relevant in horses because they are more susceptible to fungal keratitis than other domestic animal species [3,10,16,22]. Corneal trauma, prolonged topical antimicrobial or corticosteroid therapy, and impairment of local ocular defense mechanisms facilitate fungal adhesion, invasion and proliferation, allowing opportunistic fungi naturally present on the ocular surface or introduced from the environment to become pathogenic [2,3,8,23]. Because many fungal pathogens responsible for equine keratitis have also been recovered from clinically normal eyes, establishing the composition of the healthy ocular fungal community is therefore essential for distinguishing commensal or transient fungi from those associated with disease and for improving interpretation of results from molecular diagnostic methods [19].

To our knowledge, only one previous study has characterized the healthy equine ocular mycobiota using NGS, and no such a study has been performed in Purebred Lusitano horses. Therefore, the aim of the present study was to characterize the ocular surface mycobiome of clinically healthy Purebred Lusitano horses from different geographical regions and housing systems in Portugal using Oxford Nanopore long-read sequencing and conventional culture.

## 2. Materials and Methods

### 2.1. Selection of Horses

The study was approved by the Ethical Committee for Research and Teaching (CEIE) of the Faculty of Veterinary Medicine—University of Lisbon, Portugal (N/Refª 014/2020). Animal owners provided written informed consent for enrollment in the study, procedures undertaken and publication of data and images.

**Figure 1:**
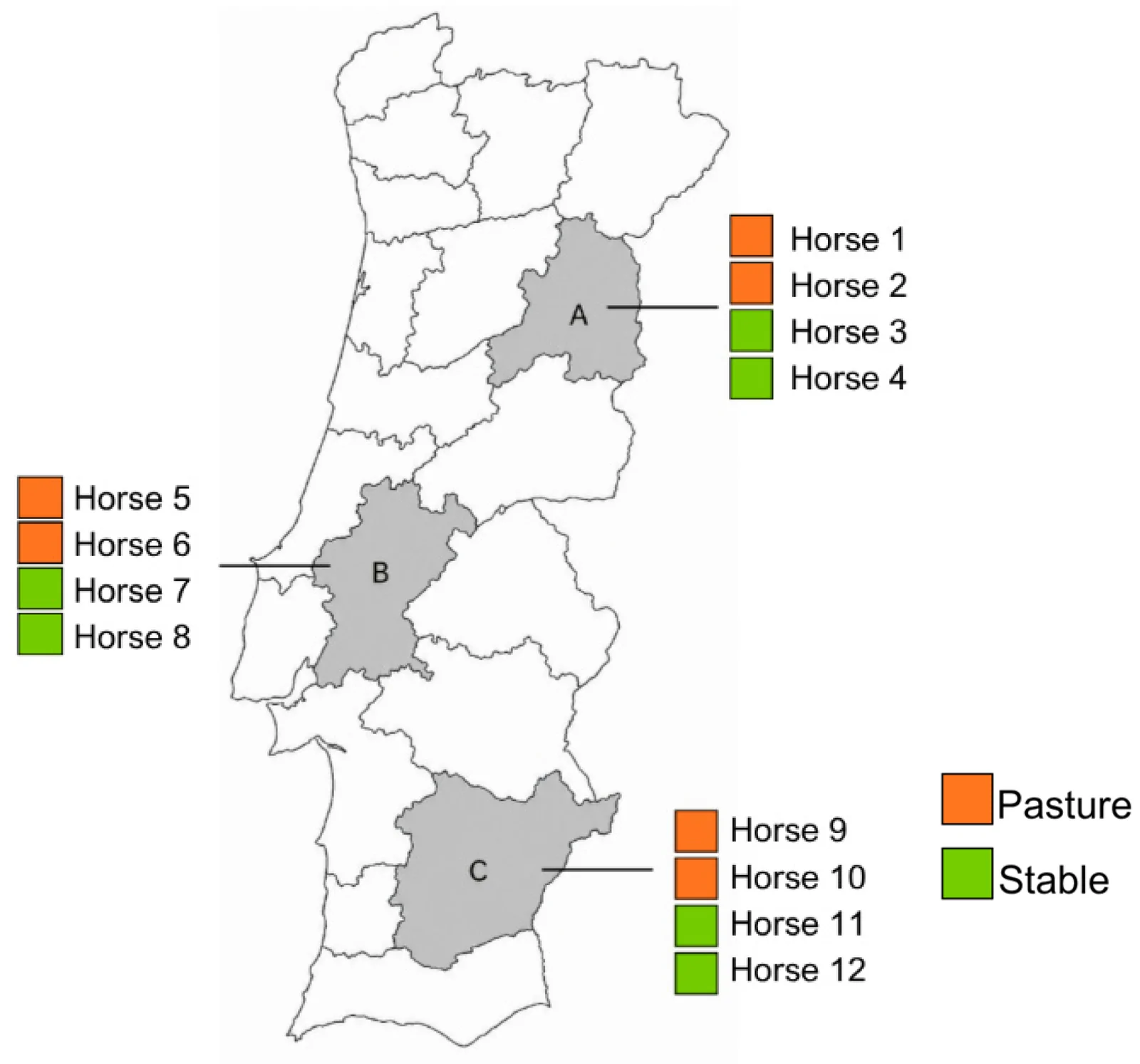
Geographical distribution of sampling locations and horses included in the study. Sampling was performed in three regions of Portugal, A, B and C, with four horses sampled per region.

Twelve clinically healthy Lusitano horses (8 mares and 4 stallions), aged between 2 and 14 years, were selected from stud farms located in three distinct regions of Portugal (Figure 1). Six horses were kept on pasture, while the other six were housed in stables. Sample collection was carried out in March 2023.

All horses underwent complete physical and ophthalmic examinations performed by a resident of the European College of Equine Internal Medicine (ECEIM). Inclusion criteria were: (1) Purebred Lusitano horses; (2) absence of ocular disease; (3) maintenance under the same housing conditions (pasture or stabling) for at least the previous six months; (4) normal findings on general clinical and ophthalmic examinations; (5) non-pregnant mares; and (6) no antibiotic or antifungal treatment within the previous 60 days.

Regarding the exclusion criteria, animals were not included in the study if they did not meet the inclusion criteria, particularly if abnormalities or values indicative of disease were detected on general physical or ophthalmic examination.

### 2.2. Sample Collection and Clinical Examination

The ophthalmic examinations included complete neuro-ophthalmic testing in which menace response, palpebral reflex, dazzle reflex, and direct and consensual pupillary light reflexes were examined in both eyes prior to any sedation. The adnexa and anterior segment were observed with slit lamp biomicroscopy (Kowa^®^ SL-15 Portable Slit Lamp, Kowa, Tokyo, Japan). Lacrimal production was assessed with the Schirmer tear test (Dina strips Schirmer-plus^®^, Luneau SAS, Chartres, France), in which samples were handled with sterile gloves to minimize the risk of sample contamination, and bilateral tonometry was conducted using Tono-Vet (Icare^®^ Tonovet Tonometer TV01, Icare Finland Oy, Vantaa, Finland) to evaluate intraocular pressure. Subsequently, a detailed examination of the eyelid margins, conjunctiva, cornea, anterior chamber, iris, and lens was performed using slit lamp biomicroscopy (Kowa^®^ SL-15 Portable Slit Lamp, Kowa, Tokyo, Japan). A volume of 0.2 mL of 1% of tropicamide (Tropicavet^®^ 10 mg/mL, VAPP—Produção e Comercialização de Produtos para Veterinária, Lda, Carnaxide, Portugal) was placed and the posterior segment—the retina and optic nerve—was examined with indirect ophthalmoscopy using a 20 D lens (Heine^®^ C-00.17.204 20D, Heine Optotechnik, Herrsching, Germany) and a handheld light source. In the end, topical fluorescein staining (I-Glo Fluorescein Sodium Ophthalmic Strips, JorVet, Loveland, CO, USA) was performed.

After sedation with detomidine hydrochloride (Domidine^®^ 10 mg/mL, Dechra Veterinary Products, Bladel, The Netherlands), conjunctival swab samples were collected from the inferior conjunctival fornix using a non-invasive ophthalmological swab method (eSwab^®^; Copan Liquid Amies preservation medium, Brescia, Italy; 490CE.A2220818). The swab was gently rotated 10 times within the fornix. In cases of swab contamination, sample collection was repeated. All samples were transported individually in their respective sterile tubes and kept refrigerated in a sealed container, and processed within 24 h of collection.

To prevent contamination or dilution, conjunctival swab samples were collected after the Schirmer tear test and before tonometry and fluorescein staining; this sampling sequence also followed that used in the previous NGS study of equine ocular mycobiota [16].

For NGS, the eye to be sampled was selected using a randomization program [24] (Sorteio Go Crie Sorteios Online Grátis [Internet]. Portugal; accessed on 3 March 2023). For mycological cultures conjunctival samples were obtained from both eyes of each animal.

### 2.3. Fungal Culture Procedures

Mycological cultures of conjunctival samples collected from both eyes of each animal were performed at the Mycology laboratory of the Faculty of Veterinary Medicine, University of Lisbon.

#### Fungal Isolation and Identification

To minimize the risk of environmental and cross-contamination during laboratory processing, each conjunctival swab was handled separately under aseptic conditions in a laminar flow cabinet, using sterile materials. Culture plates were kept open only for the time required for inoculation and were immediately closed thereafter. Samples designated for fungal culture were inoculated onto Sabouraud Dextrose Agar (SDA) (SCA—VWR Chemicals, 84,902.0500) supplemented with chloramphenicol. Following inoculation, cultures were incubated aerobically at 26 °C for 5–7 days. Petri dishes were incubated in an inverted position to minimize contamination by airborne spores during the incubation period [25].

Fungal identification was based on the assessment of both macroscopic and microscopic characteristics [17]. Regarding macroscopic evaluation, colony size, morphology, and pigmentation on both the observed and reverse surfaces of the culture medium were assessed [25].

Colony texture was classified as glabrous, velvety, yeast-like, granular, powdery, or cottony according to the characteristics of the aerial mycelium. Colony topography was described as flat, folded, wrinkled, crateriform or verrucose [26].

For microscopic examination, a portion of each colony was removed using a sterile dissecting needle. Material was collected from the actively growing region located between the center and the periphery of the colony. In the case of yeast colonies, material was collected using a sterile inoculating loop.

A drop of Lactophenol Cotton Blue (LPCB; Merck, Darmstadt, Germany 247,817) was placed on a clean glass slide, and the collected fungal material was transferred into the stain. The sample was gently fragmented and homogenized before placing a coverslip over the preparation.

Preparations were examined by light microscopy at total magnifications of ×100 and ×400. Morphological features, including hyphal septation, arrangement of reproductive structures, spore morphology, and yeast budding patterns, were recorded and used for fungal identification.

### 2.4. DNA Isolation and Processing

DNA was extracted from each swab using the MGI Easy Nucleic Acid Extraction Kit (MGI Tech Co., Ltd., Shenzhen, China) according to the manufacturer’s instructions and using the automated MGI-960 Nucleic Acid Extraction System. For some samples, DNA extraction was performed twice to obtain sufficient DNA for the subsequent amplification protocols.

DNA concentration was quantified using the Qubit dsDNA HS Assay Kit on a Qubit 4 Fluorometer (Thermo Fisher Scientific, Waltham, MA, USA), while DNA purity was assessed using a NanoDrop 1000 Spectrophotometer (Thermo Fisher Scientific, Waltham, MA, USA). The extracted DNA was stored at −20 °C until further processing.

### 2.5. PCR Amplification of the LSU (28rDNA) Region

Amplification of half of the large subunit (LSU) of eukaryotic cytoplasmatic ribosomes was performed using the fungi 28S r DNA specific primers L0R0-Fw and LR7-Rv. PCR products were evaluated by agarose gel electrophoresis and quantified using Qubit dsDNA HS Assay Kit. Samples failing amplification were re-amplified using a fresh DNA aliquot until a PCR product of sufficient quality and quantity for Oxford Nanopore sequencing was obtained. PCR amplicons were subsequently purified using the Solid-Phase Reversible Immobilization (SPRI) method with magnetic beads, as described by Stortchevoi et al. [27].

### 2.6. Nanopore Library Preparation

DNA concentration was determined using the Qubit dsDNA HS Assay Kit on a Qubit 4 Fluorometer (Thermo Fisher Scientific, Waltham, MA, USA). DNA end repair and dA-tailing were performed using the NEBNext Ultra II End Repair/dA-Tailing Module (New England BioLabs, Ipswich, MA, USA), followed by purification with Agencourt AMPure XP Beads (Beckman Coulter, High Wycombe, UK).

Libraries were prepared from 300 ng of DNA per sample using the Native Barcoding Kit 24 V14 (SQK-NBD114.24; Oxford Nanopore Technologies, Oxford, UK) according to the manufacturer’s instructions. Following library preparation, libraries were quantified, loaded onto FLO-PRO114M (R10.4.1) flow cells, and sequenced on the PromethION platform using MinKNOW v22.12.4 with the standard 72 h sequencing protocol and active channel selection enabled. The ZymoBIOMICS Microbial Community Standard was included as a sequencing/process control (Zymo Research Corp., Irvine, CA, USA).

### 2.7. Bioinformatic and Statistical Analysis

Sequencing reads were analyzed using a customized bioinformatics pipeline developed by BioISIGenomics^®^ for long-read targeted Nanopore sequencing. Quality filtering was performed using PRINSEQ-lite version 0.20.4, retaining reads with a minimum Phred quality score of 7 [28].

Taxonomic classification was performed using Kraken 2 (version 2.1.2) with the Lowest Common Ancestor (LCA) algorithm, which assigns reads based on k-mer matches to the lowest common ancestor of all genomes containing a given k-mer [29]. Taxonomic assignment was performed against a custom reference database comprising fungal sequences retrieved from the NCBI GenBank database [30] (accessed on 3 April 2023), enabling high-resolution taxonomic classification of the ocular mycobiome.

Taxonomic profiles were generated at the phylum, genus and species levels, and the relative abundance of fungal taxa was calculated for each sample.

Alpha diversity was assessed using Pielou’s evenness index. Following taxonomic classification, sequencing data were rarefied to account for differences in sequencing depth among samples. Three computational rarefaction replicates were generated for each biological sample to reduce variability associated with random subsampling. Each biological sample corresponded to one horse (*n* = 12), and the computational replicates did not represent independent biological or laboratory replicates. Therefore, the *n* values displayed in the alpha-diversity plots reflect the number of computational rarefaction replicates included in each comparison rather than the number of horses. For comparisons by geographical region, each region comprised four horses and three computational replicates per horse (*n* = 12); for comparisons by housing system, each group comprised six horses and three computational replicates per horse (*n* = 18); and for region–housing combinations, each group comprised two horses and three computational replicates per horse (*n* = 6). Differences in Pielou’s evenness were evaluated using the Kruskal–Wallis test. Pairwise comparisons were adjusted using the Benjamini–Hochberg false discovery rate (FDR) correction.

Beta diversity was assessed using Bray–Curtis dissimilarity and visualized by Principal Coordinate Analysis (PCoA). Rarefaction curves based on observed richness and the Shannon diversity index were generated to evaluate whether sequencing depth was sufficient to capture the fungal diversity present in the samples.

Taxonomic richness and the relative abundance of fungal taxa were summarized at the phylum, genus and species levels. Mean relative abundances were also calculated according to the housing system (stabled versus pasture-kept horses).

## 3. Results

All 12 horse met the inclusion criteria. All 24 examined eyes were clinically normal, and no abnormalities were identified during the ophthalmic examinations.

### 3.1. Sequence Output and Quality Assessment

Nanopore PromethION sequencing and quality filtering (minimum Phred score ≥ 7; read length of 1200–1700 bp) generated 1,351,576 high-quality reads, of which 1,233,664 were classified as eukaryotic. The mean and median of read length and quality (Read Length and Quality Reads, respectively), as well as the N50 and STDEV (standard deviation) values, are presented per sample in Table 1. Table 2 provides a summary of the number of reads classified as eukaryotic and OTUs per sample and region. A reference database was used including the NCBI Genbank reference of Fungi and the relative abundance of fungal taxa was determined for each individual sample.

### 3.2. Beta-Diversity of the Equine Ocular Mycobiome Community

**Figure 2:**
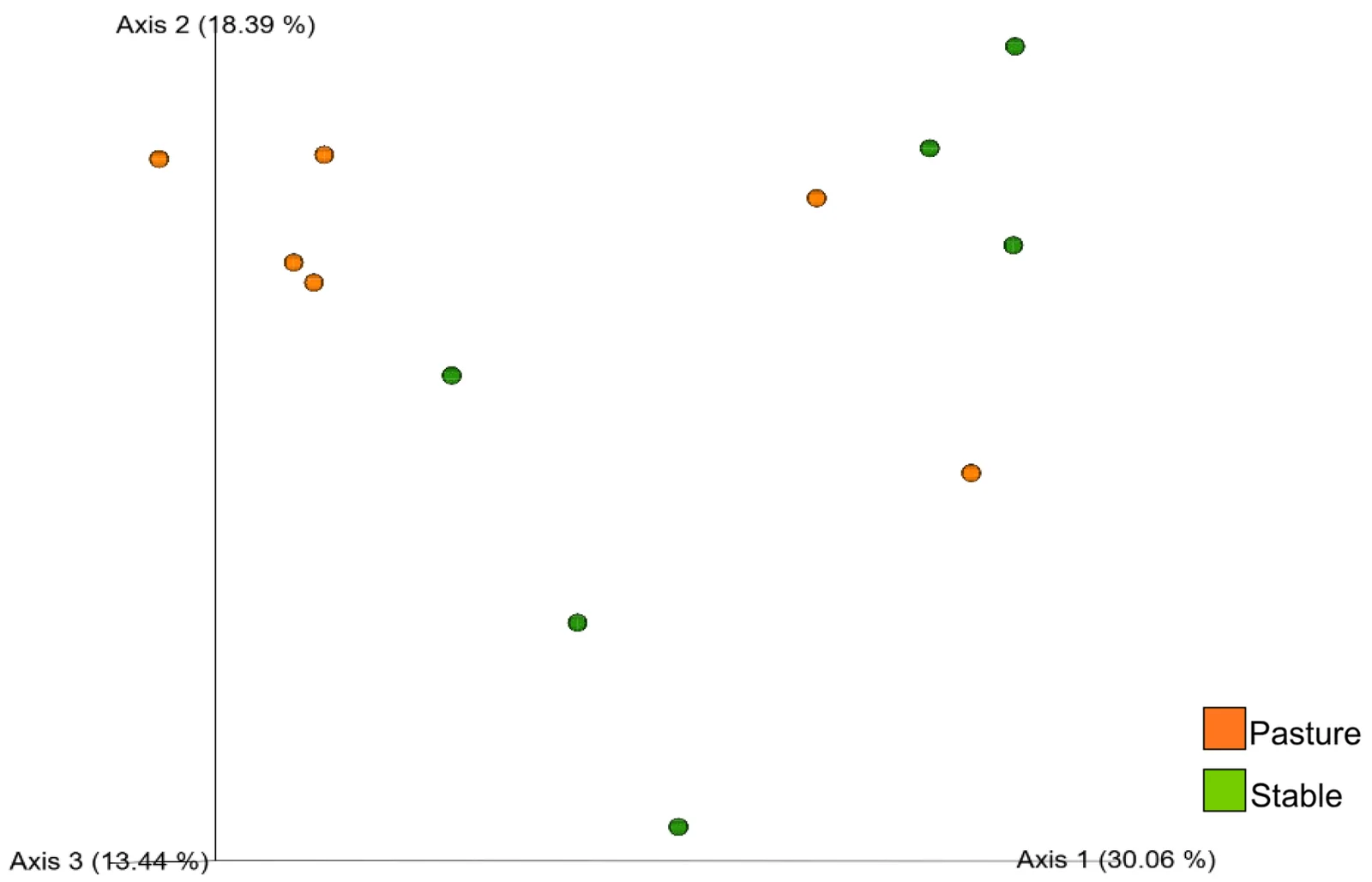
Principal Coordinate Analysis (PCoA) based on the Bray–Curtis dissimilarity index illustrates differences in fungal community composition among healthy Purebred Lusitano horses according to the housing system. Each point represents the ocular mycobiome of one horse, and the distance between points reflects the degree of compositional dissimilarity.

Beta diversity was assessed using the Bray–Curtis dissimilarity index and visualized by Principal Coordinate Analysis (PCoA). Beta diversity describes differences in fungal community composition among samples based on the relative abundance of detected taxa. In the PCoA plot, each point represents the ocular mycobiome of an individual horse, and the distance between points reflects the degree of compositional dissimilarity between samples. Visual inspection of the PCoA suggested partial overlap between pasture-kept and stabled horses, indicating that fungal community composition was not clearly separated according to the housing system (Figure 2). Although several pasture-kept horses formed a relatively compact cluster, both housing groups exhibited considerable dispersion, suggesting substantial inter-individual variation in fungal community composition.

### 3.3. Alpha-Diversity of the Equine Ocular Mycobiome Community

To evaluate fungal community diversity within individual samples, alpha diversity was assessed using Pielou’s evenness index, which measures the uniformity to the relative abundances of fungal taxa within each sample. Higher Pielou values indicate a more even distribution of taxa, whereas lower values reflect communities dominated by a smaller number of fungal taxa.

Differences in fungal community evenness among geographical regions and housing systems were evaluated using the non-parametric Kruskal–Wallis test because the data did not satisfy the assumptions of normality. Pairwise comparisons were subsequently adjusted for multiple testing using the Benjamini–Hochberg false discovery rate (FDR) correction, and adjusted *q*-values were used to determine statistical significance.

**Figure 3:**
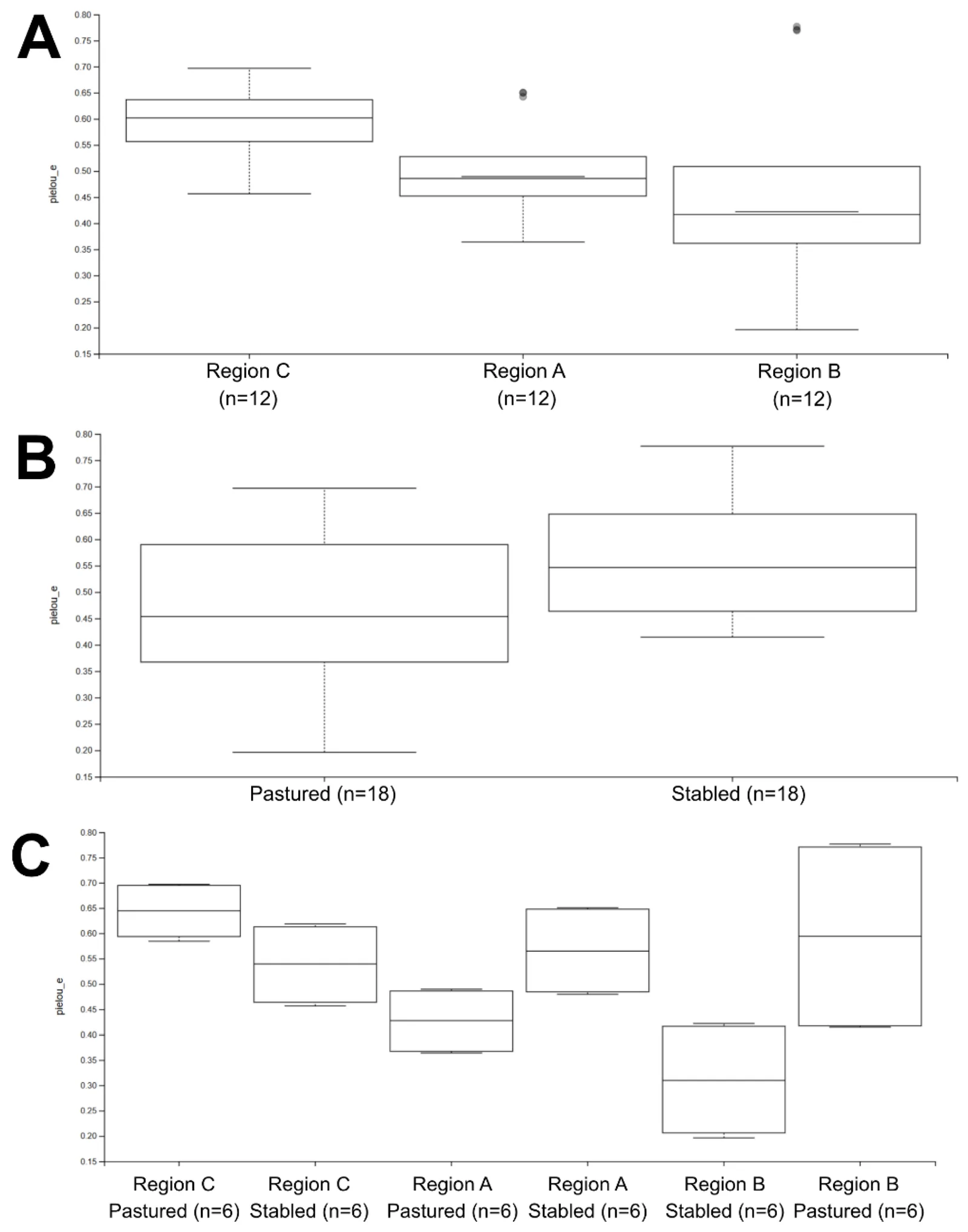
Boxplots showing fungal alpha diversity based on Pielou’s evenness index according to (**A**) geographical region, (**B**) housing system, and (**C**) the combination of geographical region and housing system. The horizontal line within each box represents the median; the box limits indicate the interquartile range (IQR); whiskers represent 1.5 × IQR; and points indicate outliers. Statistical differences were evaluated using the Kruskal–Wallis test, followed by Benjamini–Hochberg false discovery rate (FDR) correction for pairwise comparisons. *n* values represent computational rarefaction replicates rather than independent horses.

When samples from different geographical regions were analyzed independently (Figure 3A), fungal community evenness tended to be higher in horses from the region C of Portugal than those from region A and B of the country. However, the overall Kruskal–Wallis test detected no significant differences among the three regions. Pairwise comparisons similarly revealed no statistically significant differences after FDR correction, although the comparison between samples from region C and B of Portugal showed the lowest unadjusted *p*-value (*p* = 0.0377), which did not remain significant after adjustment (*q* = 0.113).

Comparison of housing systems (Figure 3B) showed slightly higher median Pielou values in stabled horses than in pasture-kept horses. Nevertheless, this difference did not reach statistical significance (Kruskal–Wallis, *p* = 0.0577) although it suggested a trend towards greater fungal community evenness in horses housed in stables.

When geographical region and housing system were analyzed jointly (Figure 3C), differences in fungal community evenness became more evident. Pairwise comparisons identified significant differences between some region–housing combinations that remained significant after FDR correction (*q* < 0.05), indicating that the fungal community evenness varied according to the combined effect of geographical location and housing system.

### 3.4. Species Richness and Diversity

Rarefaction analysis was performed suggesting that sequencing depth captured a substantial proportion of the fungal diversity present in the samples. Rarefaction curves were generated using both the observed richness metric, which estimates the number of detected taxa, and the Shannon diversity index, which incorporates both species richness and the evenness of their relative abundances.

**Figure 4:**
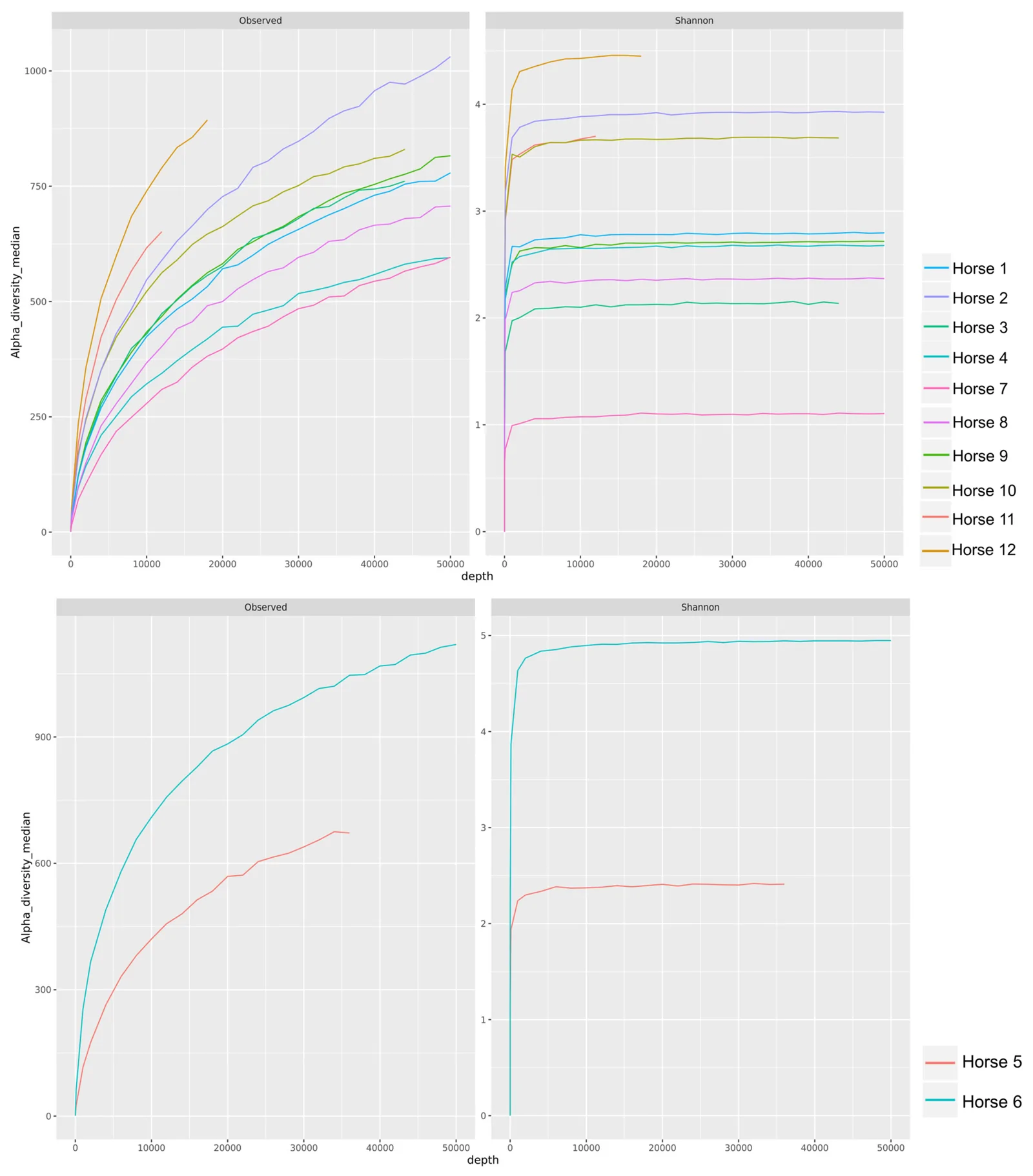
Rarefaction curves showing sequencing depth and alpha-diversity estimates of conjunctival fungal communities in twelve healthy Purebred Lusitano horses. Curves represent observed richness (number of detected taxa) and the Shannon diversity index across increasing sequencing depth. Horses were displayed in separate panels according to their sequencing depth range to improve the visualization and interpretation of the rarefaction curves.

The rarefaction curves for observed richness increased rapidly at lower sequencing depths before gradually approaching an asymptote. Likewise, Shannon diversity curves reached a stable plateau at relatively low sequencing depths (Figure 4). Collectively, these findings indicate that the sequencing effort provided adequate coverage of the fungal communities and that additional sequencing would be expected to recover relatively few additional taxa.

### 3.5. Ocular Mycobiome Composition

Nanopore sequencing of the 12 ocular samples revealed a taxonomically diverse fungal community. Individual samples contained between 4 and 9 phyla, 22 and 36 classes, 60 and 92 orders, 133 and 222 families, 209 and 435 genera and 264 and 842 OTUs. On average, each sample comprised 6.7 phyla, 28.4 classes, 69.9 orders, 170.8 families, 296.3 genera and 511.1 OTUs. Across all samples, a total of 10 phyla, 42 classes, 118 orders, 306 families, 703 genera, and 1773 OTUs were identified.

**Figure 5:**
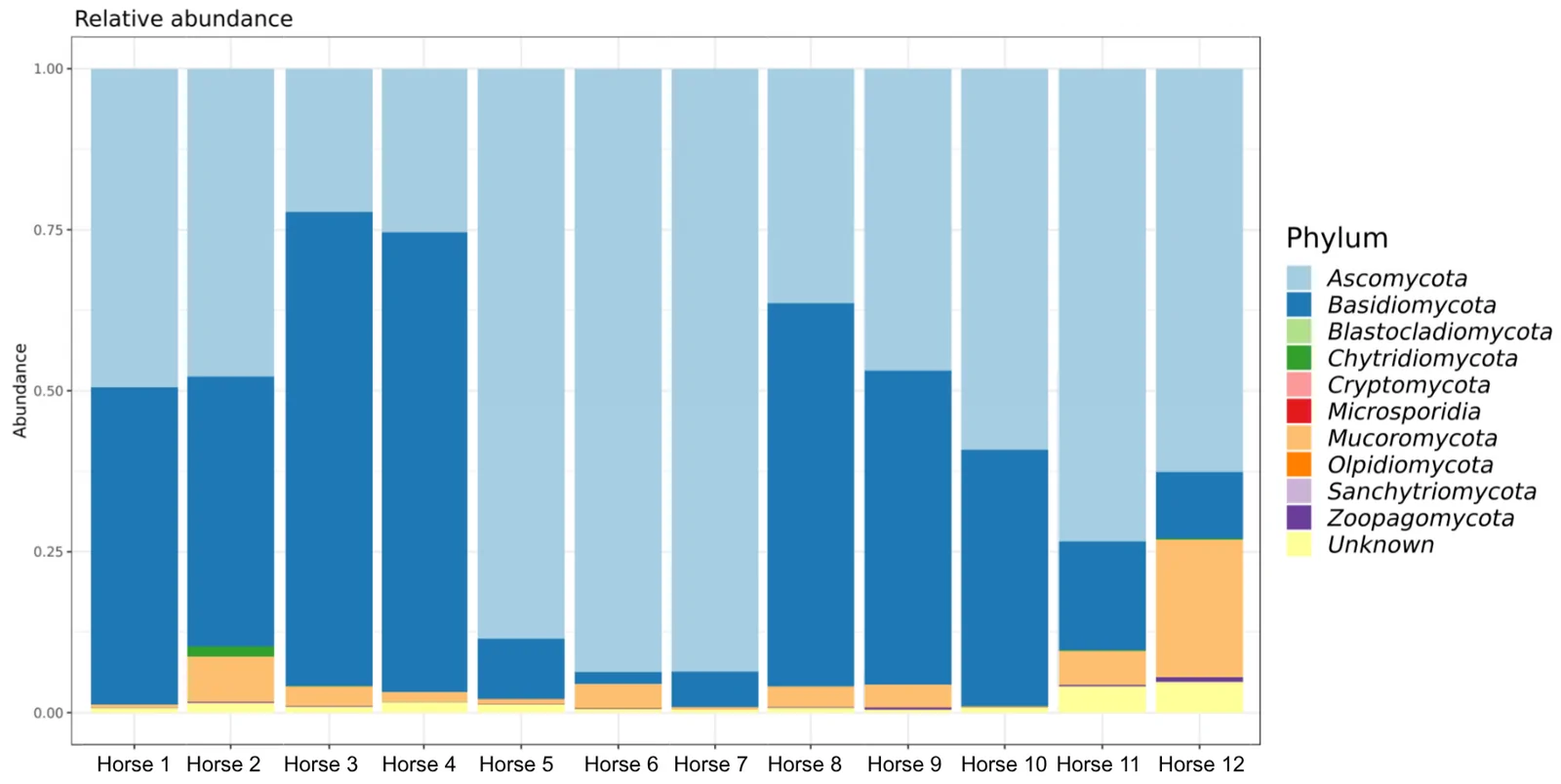
Composition of the ocular surface mycobiome in healthy horses. Relative abundance of the taxa at the phylum level. Each bar represents one sample.

At the phylum level, *Ascomycota* and *Basidiomycota* predominated across the ocular samples, with mean relative abundances of 59.14 ± 25.40% and 36.12 ± 26.35%, respectively (Table 3). *Mucoromycota* represented a smaller proportion of the fungal community (4.39 ± 6.16%) while the remaining phyla occurred at substantially lower relative abundances. The distribution of fungal phyla among individual horses is illustrated in Figure 5.

When samples were grouped according to housing system, *Ascomycota* remained the predominant phylum in both groups, with a higher mean relative abundance in stabled horses (64.88 ± 21.59%) than in pasture-kept horses (53.40 ± 29.57%). Conversely, *Basidiomycota* and *Mucoromycota* showed higher mean relative abundances in pasture-kept horses (40.27 ± 32.36% and 6.04 ± 8.32%, respectively (Table 4).

At the genus level, marked inter-individual variability was observed. The ten most abundant genera identified across the twelve horses were *Wallemia*, *Podosphaera*, *Debaryomyces*, *Aspergillus*, *Filobasidium*, *Kurtzmaniella*, *Fusarium*, *Metschnikowia*, *Spathaspora* and *Malassezia*. Their relative abundances varied considerably among individual horses, with several genera being highly abundant in individual samples but occurring at low relative abundance in others (Table 5).

**Figure 6:**
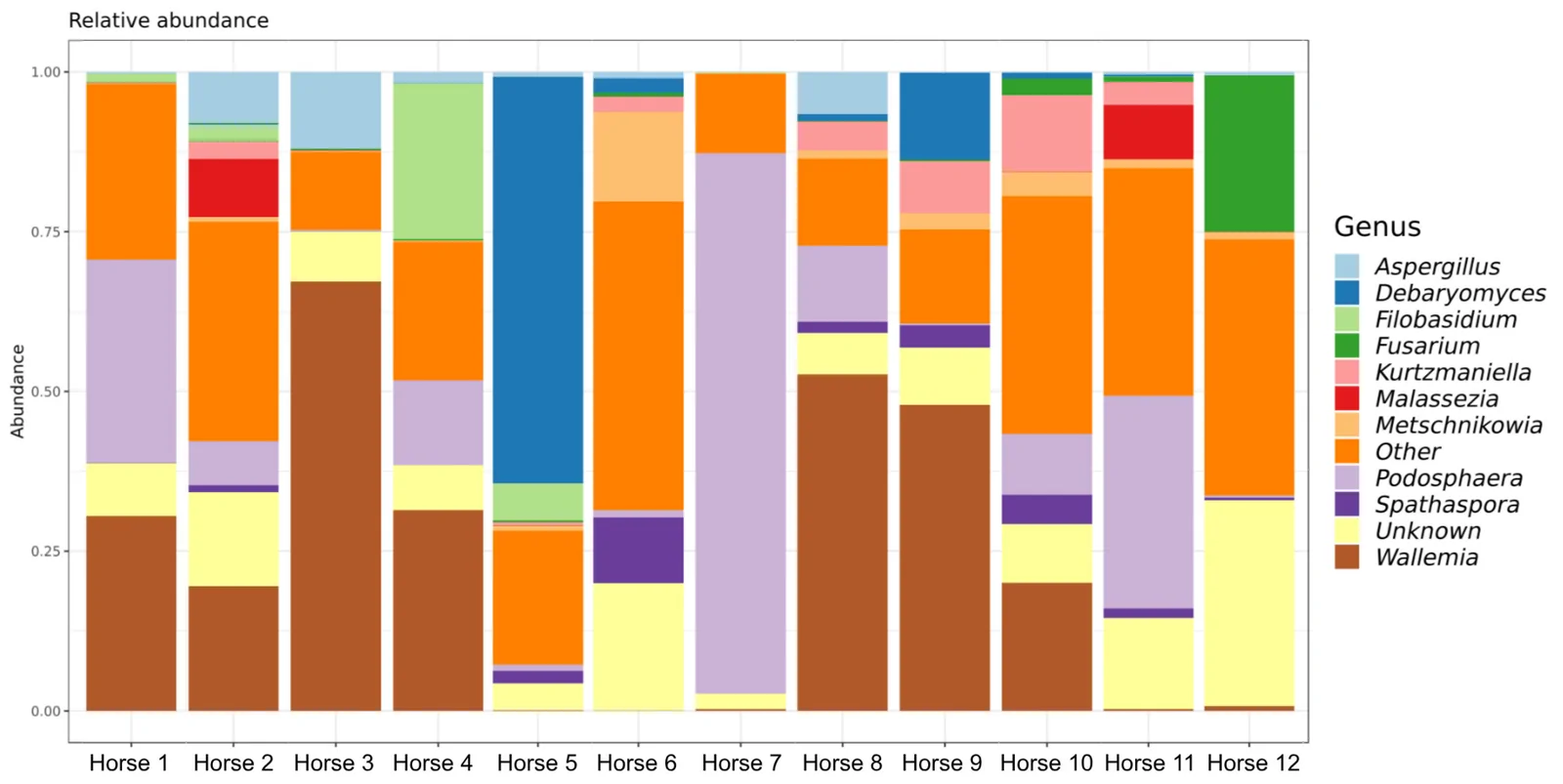
Composition of the ocular surface mycobiome in healthy horses. Relative abundance of the taxa at the genus level. Each bar represents one sample.

This heterogeneous distribution among individual horses is further illustrated in Figure 6.

**Figure 7:**
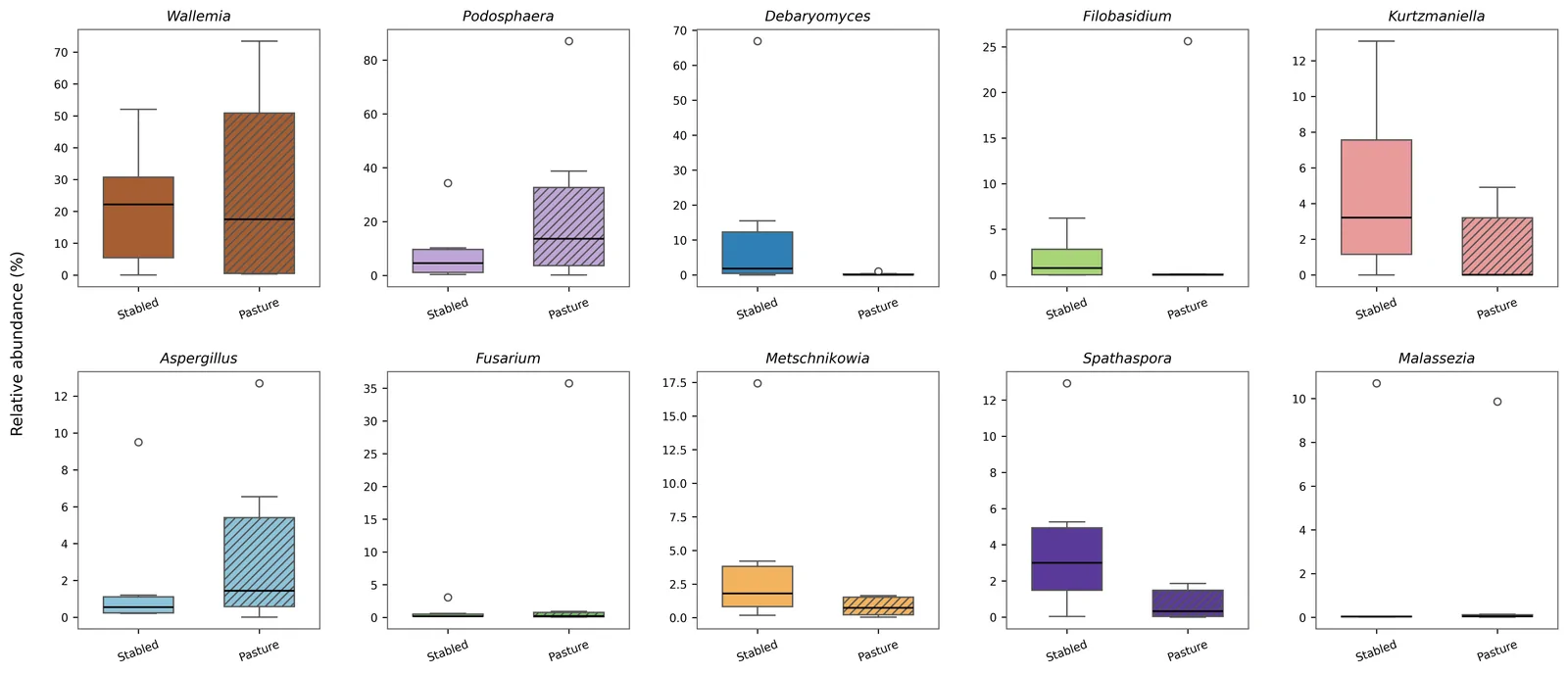
Relative abundance of the ten most abundant fungal genera identified in the healthy equine ocular mycobiome according to the housing system. Each panel represents one fungal genus. Boxplots compare stabled (*n* = 6) and pasture (*n* = 6) horses. Boxes indicate the interquartile range (IQR), the horizontal line represents the median, whiskers extend to 1.5 × IQR, and points correspond to individual ocular samples.

When genera were grouped according to housing system, differences in mean relative abundance were also observed (Table 6). *Debaryomyces*, *Kurtzmaniella* and *Metschnikowia* showed higher mean relative abundances in stabled horses, whereas *Wallemia*, *Podosphaera*, *Filobasidium*, *Aspergillus* and *Fusarium* showed higher mean relative abundances in pasture-kept horses. Considerable within-group variability was nevertheless evident for several genera. The distribution of the relative abundances of the ten most abundant fungal genera according to housing system is illustrated in Figure 7.

### 3.6. Results of Conventional Mycological Culture

Based on the conjunctival samples collected, a total of 12 fungal genera and three unidentified yeast isolates were recovered. The genera *Candida*, *Mucor* and *Aspergillus*, were the most frequently identified (Table 7).

Several fungal genera recovered by conventional culture, including *Aspergillus*, *Fusarium*, *Mucor* and *Candida*, were also detected by Nanopore sequencing.

## 4. Discussion

The present study provides the first NGS-based characterization of healthy equine ocular mycobiome using Oxford Nanopore Long Read sequencing and conventional culture from different geographical regions and housing systems in Portugal. Given the high prevalence and clinical importance of equine fungal keratitis [8,31,32,33,34,35], characterizing the normal ocular mycobiome may improve our understanding of the microbial communities inhabiting the healthy ocular surface and provide a basis for investigating factors involved in the development of ocular fungal disease.

The results revealed a highly diverse fungal community and substantial inter-individual variability, highlighting the dynamic nature of fungal communities associated with the equine ocular surface.

These findings are consistent with previous studies reporting *Ascomycota* and *Basidiomycota* as dominant components of fungal communities associated with healthy equine ocular surface [16]. Although several fungal taxa were repeatedly detected no single genus consistently dominated all samples. Instead, individual horses frequently exhibited distinct community profiles characterized by varying abundances of *Wallemia*, *Podophaera*, *Debaryomyces*, *Fusarium*, *Filobasidium* and *Aspergillus*. This high degree of heterogeneity indicates that the equine ocular mycobiome is highly variable, even among horses maintained under similar environmental conditions, suggesting a dynamic fungal assemblage rather than a stable resident community. These observations are consistent with previous sequencing-based studies of healthy equine eyes. Walsh et al. [16] demonstrated that clinically healthy horses harbor complex fungal communities that are considerably more diverse than previously recognized through culture-based techniques. Similarly, studies of the human ocular microbiome have shown that the ocular surface represents a low biomass ecosystem continuously influenced by microorganisms originating from surrounding environment [36,37].

Our group’s clinical experience in managing equine ocular disease suggested an apparent increase in the frequency of fungal isolation, particularly from horses with corneal ulceration. This observation led us to hypothesize that environmental conditions may influence the ocular mycobiome, with potential implications for the development and clinical course of fungal keratitis. Supporting this hypothesis and using NGS, Walsh et al. [16] demonstrated significant differences in both alpha and beta diversity between pasture-kept and stabled horses, with pasture-kept animals exhibiting greater richness, abundance, and taxonomic diversity. Our findings contrast with those reported by Walsh et al. [16], as our diversity analyses revealed only a limited influence of housing conditions on fungal community structure. Although a trend towards differences between pastured and stabled horses was observed, statistical significance was not reached following correction for multiple testing. This finding suggests that housing condition alone may not be a major determinant of fungal diversity. Horses maintained under both management systems are continuously exposed to environmental fungi through multiple sources, including feed, bedding materials, dust, vegetation, and airborne fungal particles [13,15,38,39]. This shared and continuous environmental exposure may partly explain the limited differences observed between housing systems in the present study.

Although overall alpha diversity did not differ significantly between samples from animals from different housing systems, differences in taxonomic composition were observed. *Wallemia* was among the most abundant genera identified, but while pasture-kept horses were predominantly associated with *Wallemia mellicola*, stabled horses harbored *Wallemia melicola* and *Wallemia hedeare*. Similarly, *Podosphaera*, a dominant genus detected in the present study and a recognized plant-associated powdery mildew fungus, predominated in pasture-kept horses, accounting for 25.65 ± 33 (19%) of all fungi detected in this group of animals in comparison with only 9.19 ± 12.9 (6%) in stabled horses. The latter values for *Podosphera* are skewed by one pasture horse that accounted for 87.05% of this genus. In contrast, stabled horses exhibited a more heterogeneous fungal community characterized by a lower dominance of individual genera. Together, these observations suggest that the healthy equine ocular mycobiome is primarily shaped by environmental exposure. Members of the genus *Wallemia* are commonly associated with airborne dust, hay, feed and other dry environmental substrates and are recognized as highly xerotolerant fungi [9,14,15]. Likewise, *Podosphaera* is closely associated with vegetation, whereas other frequently detected genera, including *Fusarium*, *Aspergillus*, *Debaromyces* and *Filobasidium*, are widely distributed environmental fungi associated with soil, vegetation, and other organic substrates, supporting the hypothesis that their detection on the equine ocular surface may at least partly reflect continuously environmental exposure [40,41,42].

Interestingly, total eukaryotic read counts were also higher in stabled horses than in pastured animals. Although total eukaryotic read counts differed among samples, sequencing read abundance should not be interpreted as a direct measure of fungal burden because it may be influenced by amplification and sequencing efficiency. Nevertheless, caution is warranted when interpreting these findings because substantial inter-individual variation was observed, particularly in one pastured horse (Horse 7) from one region (B), which accounted for a disproportionately large number of sequencing reads.

Although alpha diversity did not differ significantly among animals from different geographical regions (*p* = 0.0732) or between housing systems when analyzed independently (*p* = 0.057), a significant effect was observed when both variables were considered together (*p* = 0.0039). This finding suggests that the influence of housing on the equine ocular mycobiome is context-dependent and may vary according to the local environmental conditions. Similarly, beta-diversity analyses demonstrated that geographical location was associated with a greater proportion of fungal community variation than housing alone. Although some pairwise regional comparisons did not remain significant after correction for multiple testing, consistent differences were observed, particularly between the South and Center regions. These findings suggest that local environmental conditions associated with geographical location, including climate, vegetation, land use, and environmental fungal reservoirs, may interact with management practices to shape the composition of the healthy equine ocular mycobiota. Similar environmental drivers of fungal community composition have been reported in large-scale ecological studies.

Several fungal genera commonly associated with equine fungal keratitis, including *Aspergillus*, *Fusarium* and *Penicillium*, were detected in healthy horses irrespective of housing system [15,43,44,45]. However, no marked differences in their relative abundances were observed between stabled and pastured horses. These findings indicate that the ocular surface is not sufficient for disease development and support the concept that fungal keratitis is a multifactorial disease requiring additional predisposing factors, such as corneal trauma, impairment of local immune defenses, tear film disruption, or environmental stressors [8,11].

The fungal community composition differed from that reported by Walsh et al. [16], who identified *Leptosphaerulina*, unclassified *Pleosporaceae*, *Cladosporium* and *Alternaria* in pasture-kept horses. These discrepancies are likely attributable to differences in geographical location, climatic conditions, husbandry practices, sampling season, or environmental fungal exposure.

Among the detected genera, *Aspergillus* and *Fusarium* deserve particular attention because they are the principal etiological agents of equine fungal keratitis and have previously been isolated from healthy equine eyes [5,10,13]. In the present study, *Aspergillus* was identified in all geographical regions by NGS and recovered by conventional fungal culture, whereas *Fusarium* species were detected in both housing systems. Similar observations have been reported in other species, where potentially pathogenic fungi may be present on the healthy ocular surface without causing disease [46]. Collectively, these findings reinforce that exposure to potentially pathogenic fungi is a normal feature of the healthy equine ocular surface and that disease results from a complex interaction between the microorganism, the host, and environmental factors [11,16,39].

The limited agreement between NGS and conventional fungal culture observed in the present study was expected and reflects the inherent differences between these methodologies. Conventional culture selectively detects viable fungi capable of growing under laboratory conditions and therefore underestimates fungal diversity, as many fungal taxa are difficult or impossible to cultivate using standard microbiological techniques [47,48]. This limitation has also been demonstrated in previous studies on healthy horses, which reported fungal culture positivity ranges from 13% to 76.8% depending on the study population and culture methodology [6,15].

In contrast, NGS identifies fungi through their genetic material and is therefore capable of detecting a substantially broader spectrum of taxa, including organisms present in low abundance or those that are not readily culturable [20,49]. Similar observations have been reported in both veterinary and human ophthalmology, where molecular sequencing consistently reveals a more diverse ocular mycobiota than culture-based methods and has identified numerous fungal taxa not previously recognized by conventional microbiological techniques [48,50,51].

It is important to note that sequencing-based approaches also have important limitations. Because sequencing detects fungal DNA rather than viable organisms, it cannot distinguish between active colonization and transient or non-viable fungal particles, which is particularly relevant when studying the healthy ocular surface [52]. Furthermore, sequencing results may be influenced by technical artifacts [49]. In addition, the accuracy of taxonomic assignment depends on the completeness and curation of the reference database, which may influence the classification of some fungal taxa. Consequently, NGS and conventional fungal culture should be regarded as complementary rather than competing techniques, each providing distinct but valuable information for characterizing the equine ocular mycobiota.

Several limitations of the present study should be acknowledged. First, amplicon sequencing detects fungal DNA regardless of viability and therefore cannot distinguish active colonization from transient environmental deposition. In addition, the small number of horses within each geographical region and housing subgroup limits the ability to disentangle the independent effects of geographical location and housing conditions. The relatively small number of animals included in each subgroup limited the statistical power to detect subtle ecological differences and reduced the representativeness of the findings for the broader Lusitano horse population in Portugal. Although the use of sterile strips and gloves minimized the risk of contamination, a minor influence of the Schirmer tear test on subsequent microbiological sampling cannot be completely excluded. Furthermore the interval between sample collection and fungal culture processing may have influenced culture recovery, particularly for fast-growing yeasts such as *Candida* spp. Finally, environmental control samples were not included, preventing direct assessment of environmental fungal reservoirs and their contribution to the ocular mycobiota.

Future studies including larger cohorts, environmental sampling, and longitudinal monitoring across different seasons would improve our understanding of the temporal stability of the equine ocular mycobiota and its relationship with environmental exposure. Future research should apply the same methodological approach to horses with ocular disease and compare their ocular mycobiota with that of healthy horses. Such studies could help identify disease-associated changes in fungal community composition and diversity and clarify the potential role of microbial dysbiosis in the development and progression of ocular disease.

## 5. Conclusions

This study provides the first NGS-based characterization of the healthy ocular mycobiome of Purebred Lusitano horses in Portugal. The results demonstrated that the equine ocular surface harbors a diverse fungal community dominated by environmentally associated taxa. The marked inter-individual variability is consistent with a substantial influence of continuous environmental exposure on the equine ocular fungal community. Taxa such as *Wallemia* sp., *Podosphaera* sp., *Debaryomyces* sp., *Fusarium* sp. and *Filobasidium* sp. were frequently detected and likely reflect continuous contact with airborne particles, vegetation, and environmental reservoirs.

The housing system exerted only a limited influence on fungal diversity, whereas geographical location contributed more substantially to variation in fungal community composition. The significant interaction observed between geographical location and housing system further supports the importance of local environmental conditions in shaping the healthy equine ocular mycobiome.

Collectively, these findings establish an important baseline for future studies investigating alterations of the equine ocular mycobiome associated with fungal keratitis and other ocular surface diseases. They also demonstrate the value of long-read Oxford Nanopore sequencing for the comprehensive characterization of the healthy equine ocular mycobiome, providing a reference framework for future studies on ocular fungal dysbiosis and equine ocular health.

Although NGS substantially expands the detection of fungal taxa, conventional culture remains indispensable because it confirms microbial viability, permits phenotypic identification, and allows antifungal susceptibility testing. Consequently, both approaches should be regarded as complementary methods for investigating the equine ocular mycobiome rather than mutually exclusive techniques.

## Figures and Tables

**Table 1:** Quality of sequence analysis. Read length and N50 are expressed in base pairs (bps). N50 represents the length of the shortest sequenced fragment for which fragments of that length or longer collectively account for at least 50% of the total sequenced bases; STDEV (standard deviation).

Sample	Read Length (bps)		Quality Reads		N50 (bps)	STDEV
	Mean	Median	Mean	Median		
Horse 1	1535.5	1562.0	14.6	14.9	1563.0	72.6
Horse 2	1454.0	1533.0	14.6	14.8	1538.0	144
Horse 3	1500.2	1554.0	14.7	14.9	1558.0	115.4
Horse 4	1520.9	1563.0	14.7	15.0	1565.0	107.6
Horse 5	1507.5	1540.0	14.5	14.7	1540.0	88.5
Horse 6	1489.9	1537.0	14.4	14.7	1538.0	111.6
Horse 7	1542.9	1566.0	14.5	14.8	1566.0	69.1
Horse 8	1488.2	1540.0	14.7	15.0	1545.0	123.2
Horse 9	1496.6	1542.0	14.7	15.0	1544.0	118.8
Horse 10	1522.3	1543.0	14.5	14.8	1544.0	77.7
Horse 11	1431.7	1505.0	14.5	14.8	1515.0	144.4
Horse 12	1341.4	1257.0	14.6	14.9	1260.0	140.3

**Table 2:** Number of eukaryotic reads and OTUs per sample and region.

Region A			Region B			Region C		
Samples	Reads (nº)	OTUs (nº)	Samples	Reads (nº)	OTUs (nº)	Samples	Reads (nº)	OTUs (nº)
Horse 1	217,527	231	Horse 5	36,419	221	Horse 9	80,288	233
Horse 2	169,383	297	Horse 6	77,050	425	Horse 10	44,148	323
Horse 3	44,985	229	Horse 7	418,900	128	Horse 11	13,456	394
Horse 4	59,366	172	Horse 8	53,716	185	Horse 12	18,426	486

**Table 3:** Relative abundance (%) of the eight most abundant fungal phyla across the ocular samples of twelve healthy horses (*n* = 12).

Relative Abundance (%)													
Phylum	Horse												Mean Relative Abundance (%) ± SD
	1	2	3	4	5	6	7	8	9	10	11	12	
*Ascomycota*	49.86	47.77	21.83	25.75	89.82	94.33	93.93	36.64	47.60	59.93	76.54	65.72	59.14 ± 25.40
*Basidiomycota*	49.53	43.04	74.71	72.73	9.27	1.67	5.54	60.33	48.43	39.86	17.62	10.71	36.12 ± 26.35
*Mucoromycota*	0.59	7.30	3.28	1.38	0.75	3.89	0.52	2.91	3.71	0.15	5.50	22.66	4.39 ± 6.16
*Chytridiomycota*	0.01	1.77	0.02	0.03	0.03	0.01	0.00	0.00	0.01	0.01	0.07	0.16	0.18 ± 0.50
*Zoopagomycota*	0.01	0.11	0.16	0.09	0.02	0.10	0.01	0.09	0.25	0.01	0.23	0.70	0.15 ± 0.19
*Cryptomycota*	0.00	0.00	0.01	0.00	0.10	0.00	0.00	0.01	0.00	0.01	0.00	0.01	0.01 ± 0.03
*Microsporidia*	0.00	0.01	0.01	0.02	0.00	0.00	0.00	0.01	0.00	0.01	0.02	0.02	0.01 ± 0.01
*Olpidiomycota*	0.00	0.00	0.00	0.00	0.01	0.01	0.00	0.01	0.00	0.01	0.02	0.02	0.01 ± 0.01

**Table 4:** Relative abundance (%) of the identified fungal phyla sequenced from the equine ocular surface of healthy horses in different housing environments.

	Stabled Horses	Pastured Horses
Phylum	Mean Relative Abundance ± SD (%)	Mean Relative Abundance ± SD (%)
*Ascomycota*	64.88 ± 21.59	53.40 ± 29.57
*Basidiomycota*	31.97 ± 20.96	40.27 ± 32.36
*Mucoromycota*	2.73 ± 2.77	6.04 ± 8.32
*Chytridiomycota*	0.31 ± 0.72	0.05 ± 0.06
*Zoopagomycota*	0.08 ± 0.10	0.21 ± 0.25
*Cryptomycota*	0.02 ± 0.04	0.00 ± 0.00
*Microsporidia*	0.00 ± 0.01	0.01 ± 0.01
*Olpidiomycota*	0.00 ± 0.01	0.01 ± 0.01

**Table 5:** Relative abundance (%) of the ten most abundant fungal genera across the ocular samples of twelve healthy horses (*n* = 12).

Relative Abundance (%)													
*Genus*	Horse												Mean Relative Abundance (%) ± SD
	1	2	3	4	5	6	7	8	9	10	11	12	
*Wallemia*	33.44	22.69	73.47	34.01	0.08	0.04	0.34	56.51	52.05	21.64	0.37	0.97	24.63 ± 24.63
*Podosphaera*	34.28	7.94	0.19	14.49	1.07	1.23	87.05	12.78	0.35	10.25	38.77	0.63	17.42 ± 24.43
*Debaryomyces*	0.00	0.26	0.02	0.02	66.91	2.72	0.01	1.02	15.50	0.94	0.38	0.04	7.32 ± 18.45
*Filobasidium*	1.52	3.26	0.01	25.63	6.23	0.01	0.11	0.00	0.01	0.00	0.02	0.00	3.07 ± 7.05
*Kurtzmaniella*	0.00	3.28	0.01	0.01	0.49	3.14	0.00	4.91	8.99	13.10	4.27	0.01	3.18 ± 4.02
*Aspergillus*	0.24	9.50	12.70	1.98	0.86	1.20	0.01	6.55	0.24	0.21	0.47	0.91	2.91 ± 4.09
*Fusarium*	0.16	0.17	0.30	0.11	0.19	0.62	0.05	0.10	0.19	3.06	0.94	35.76	3.47 ± 9.77
*Metschnikowia*	0.18	0.78	0.19	0.28	0.96	17.44	0.04	1.19	2.65	4.21	1.63	1.64	2.60 ± 4.62
*Spathaspora*	0.04	1.30	0.06	0.03	2.04	12.93	0.00	1.86	3.96	5.27	1.78	0.60	2.49 ± 3.52
*Malassezia*	0.03	10.70	0.05	0.04	0.03	0.05	0.01	0.03	0.02	0.02	9.86	0.14	1.75 ± 3.82

**Table 6:** Relative abundance (%) of the identified fungal genera sequenced from the equine ocular surface of healthy horses in different housing environments.

	Stabled Horses	Pastured Horses
Genus	Mean Relative Abundance ± SD (%)	Mean Relative Abundance ± SD (%)
*Wallemia*	21.66 ± 19.98	27.61 ± 32.17
*Podosphaera*	9.19 ± 12.96	25.65 ± 33.19
*Debaryomyces*	14.39 ± 26.39	0.25 ± 0.40
*Filobasidium*	1.84 ± 2.51	4.29 ± 10.45
*Kurtzmaniella*	4.83 ± 5.16	1.53 ± 2.37
*Aspergillus*	2.04 ± 3.67	3.77 ± 4.98
*Fusarium*	0.73 ± 1.15	6.21 ± 14.48
*Metschnikowia*	4.37 ± 6.57	0.83 ± 0.74

**Table 7:** Summarizes the absolute frequency (AF) and relative frequency (RF) of each fungal isolate (*n* = 24) from conventional fungal culture-based technique.

Fungal Group	Taxon	Absolute Frequency (*n*)	Relative Frequency (%)
Filamentous fungi	*Mucor* spp.	14	23
	*Aspergillus* spp.	12	20
	*Alternaria* sp.	2	3
	*Cladosporium* sp.	2	3
	*Geotrichum* sp.	2	3
	*Penicillium* sp.	2	3
	*Fusarium* sp.	1	2
	*Phialophora* sp.	1	2
	*Trichoderma* sp.	1	2
Yeasts	*Candida* sp.	16	27
	*Rhodotorula* sp.	3	5
	Non-identified yeast	3	5
	*Trichosporon* sp.	1	2

## Data Availability

The raw sequencing data generated in this study have been deposited in the NCBI Sequence Read Archive (SRA) under BioProject accession number PRJNA1496331.

## Abbreviations

The following abbreviations are used in this manuscript:

CEIE	Ethical Committee for Research and Teaching
DNA	Deoxyribonucleic Acid
dsDNA	Double-Stranded DNA
gDNA	Genomic DNA
rDNA	Ribosomal DNA
ECEIM	European College of Equine Internal Medicine
FDR	False Discovery Rate
IQR	Interuartile Range
LPCB	Lactophenol Cotton Blue
LSU	Large Subunit
NCBI	National Center for Biotechnology Information
NGS	Next-Generation Sequencing
OTUs	Operational Taxonomic Units
PCoA	Principal Coordinate Analysis
PCR	Polymerase Chain Reaction
SCA	Sabouraud Cloranfenicol Agar
SDA	Sabouraud Dextrose Agar
SPRI	Solid-Phase Reversible Immobilization
